# Do Complex Pathologies Remain a Challenge in Minimally Invasive Mitral Valve Surgery?

**DOI:** 10.31083/RCM44923

**Published:** 2025-10-22

**Authors:** Martina Dini, Leonard Pitts, Matteo Montagner, Serdar Akansel, Emilija Miskinyte, Dustin Greve, Stephan Jacobs, Volkmar Falk, Jörg Kempfert, Markus Kofler

**Affiliations:** ^1^Department of Cardiothoracic and Vascular Surgery, Deutsches Herzzentrum der Charité (DHZC), 13353 Berlin, Germany; ^2^Charité – Universitätsmedizin Berlin, Corporate Member of Freie Universität Berlin and Humboldt-Universität zu Berlin, 10117 Berlin, Germany; ^3^DZHK (German Centre for Cardiovascular Research), Partner Site Berlin, Germany; ^4^Translational Cardiovascular Technologies, Institute of Translational Medicine, Department of Health Sciences and Technology, Swiss Federal Institute of Technology (ETH), 8093 Zurich, Switzerland

**Keywords:** minimally invasive cardiac surgery, minimally invasive mitral valve repair, mitral valve endocarditis, mitral annulus calcification, Barlow's disease

## Abstract

Minimally invasive mitral valve repair (MI-MVr) is the preferred treatment approach in experienced centers for mitral valve disease (MVD), offering reduced surgical trauma and fast recovery. However, limited operative exposure and increased procedural complexity can represent a challenge in complex MVD. This narrative review provides an overview of current literature on clinical outcomes of MI-MVr in challenging MVD scenarios, such as mitral valve (MV) endocarditis, annulus calcification, and mitral annular disjunction, in the context of myxomatous MVD. Despite the complex anatomy and MVD, MI-MVr is non-inferior in long-term outcomes in treating MV endocarditis, MV calcification, and myxomatous MVD with mitral annular disjunction. Nonetheless, careful patient selection and referral to high-volume centers, where surgeons with expertise in MI-MVr operate, are key elements for achieving a durable, patient-tailored repair with an optimal long-term outcome in treating complex MVD.

## 1. Introduction 

The prevalence of valvular heart disease is around 2.5% [1], with mitral regurgitation (MR) being the most common disease [1, 2, 3] and the second most frequent indication for valve surgery in Europe [4, 5].

MR is mostly caused by primary and secondary mitral valve disease (MVD) [6]. Primary, or degenerative, MVD refers to a spectrum of conditions caused by morphological changes in the connective tissue of the mitral valve (MV) with consequential structural lesions that prevent the normal function of the mitral apparatus [7]. Fibroelastic deficiency and Barlow’s disease are the two dominant forms of degenerative MVD [7].

Secondary, or functional, MVD can be categorized in two main groups: ventricular MVD, which originates from leaflet tethering by geometric remodeling of the left ventricle, usually resulting from scar formation after myocardial infarction or dilated cardiomyopathy [8]; atrial MVD, which presents with preserved ventricular geometry and function, but with a mitral annular enlargement associated with left atrial dilatation in the setting of chronic atrial fibrillation or heart failure with preserved ejection fraction [9, 10].

Current European Society of Cardiology/European Association for Cardio-Thoracic Surgery (ESC/EACTS) guidelines for the management of valvular heart disease [11] recommend surgical treatment of chronic degenerative MR in symptomatic patients with severe primary MR (low operative risk and expected durable results) or with signs of ongoing left ventricular remodeling regardless of the symptomatic status (left ventricular end systolic diameter ≥40 mm and/or left ventricular ejection fraction <60%). Surgical treatment of chronic functional MR is recommended in symptomatic patients despite optimal pharmacological therapy and in patients undergoing other cardiac surgery procedures; meanwhile, transcatheter edge-to-edge repair serves as an alternative in selected cases [11]. Urgent surgery is indicated in patients with acute severe MR [11].

Mitral valve repair (MVr) represents the gold standard surgical therapy for MR [2, 12]. MVr aims to restore and preserve MV leaflet mobility, create a large surface of leaflet coaptation, and remodel the mitral annulus to provide an optimal and stable orifice area [2].

MVr is associated with high patient satisfaction, reduced hospital stays, low perioperative morbidity and mortality rates [4], as well as excellent long-term outcomes and freedom from reoperation [13, 14, 15, 16].

The surgical technique for repairing the MV should be selected according to the specific valve pathology and the anatomy of the patient. A careful investigation of the patient and an appropriate discussion in the Heart Team are fundamental steps in the preoperative planning of MVr [17].

Accordingly, minimally invasive surgery (MIS)—defined by a sternum-sparing approach—has been established as the gold standard for MVr [18] and represents a routine operative strategy for treating MR in specialized centers with corresponding expertise.

MIS is associated with a decrease in surgical trauma, fewer blood transfusions, less pain, shorter ventilation time, reduced length of stay in the intensive care unit, shorter hospitalization time, earlier return to normal activities, lower risk of infections, and cosmetic improvements when compared to conventional approaches [2, 19, 20].

However, minimally invasive mitral valve repair (MI-MVr) may be technically more challenging to learn and perform than MVr through median sternotomy. Common concerns for a minimally invasive approach include a limited operative space, an extended distance from the chest wall to the MV, the need for specialized equipment and operative tools, restricted exposure of the surrounding structures, as well as the need for special surgical training [21], which possesses a steep learning curve [22]. Nonetheless, MI-MVr has been demonstrated to be a feasible and safe option, especially in high-volume institutions that guarantee high and durable repair rates [4].

**Fig. 1.**
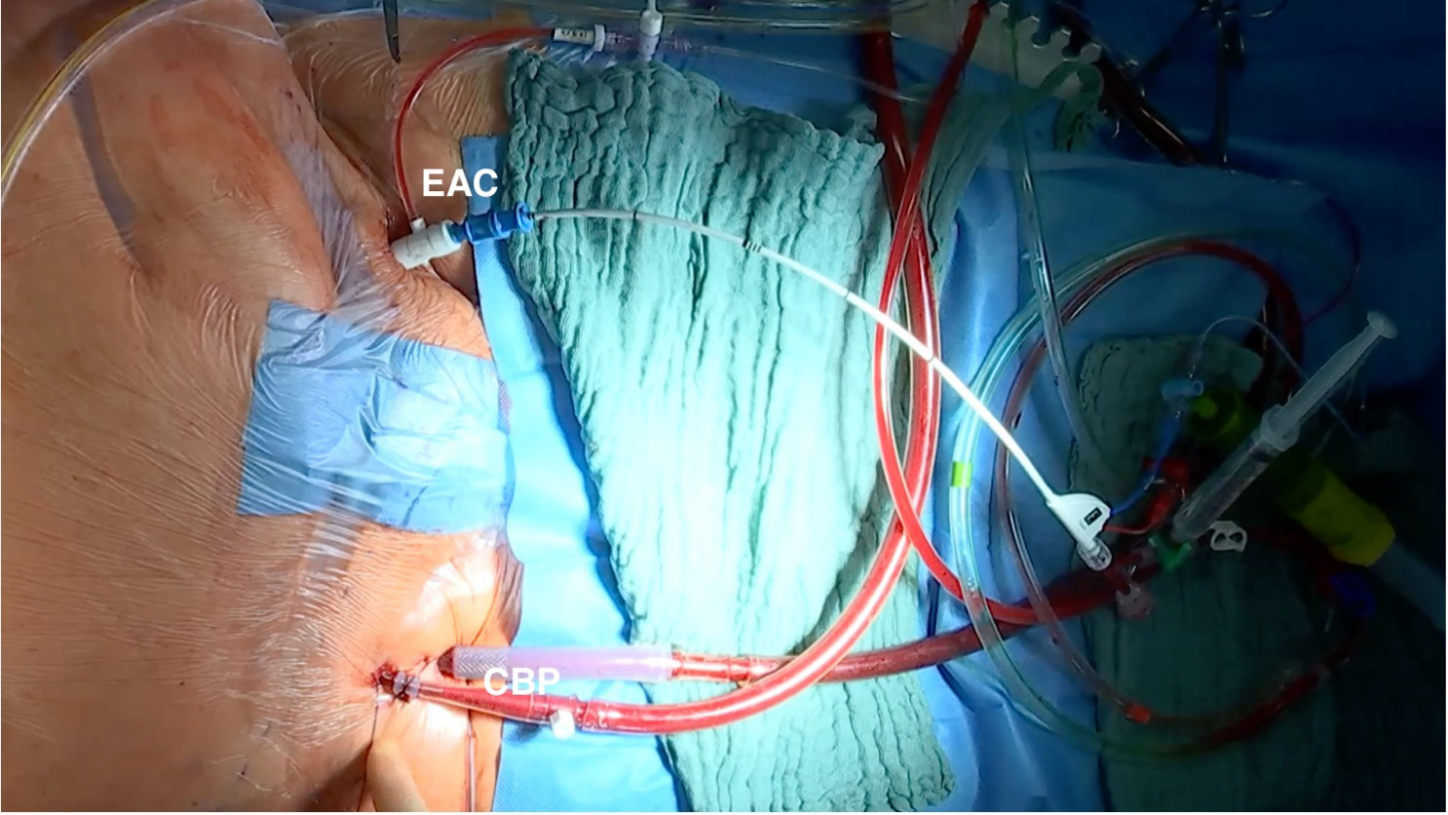
**Cardiopulmonary bypass and endoaortic clamp inguinal setup**. The patient is connected to cardiopulmonary bypass by percutaneous cannulation of the right femoral artery and vein. An aortic endoclamp is percutaneously placed through the left femoral artery, which allows aortic cross-clamping, antegrade cardioplegia administration, and aortic root venting. EAC, endoaortic clamp; CBP, cardiopulmonary bypass.

**Fig. 2.**
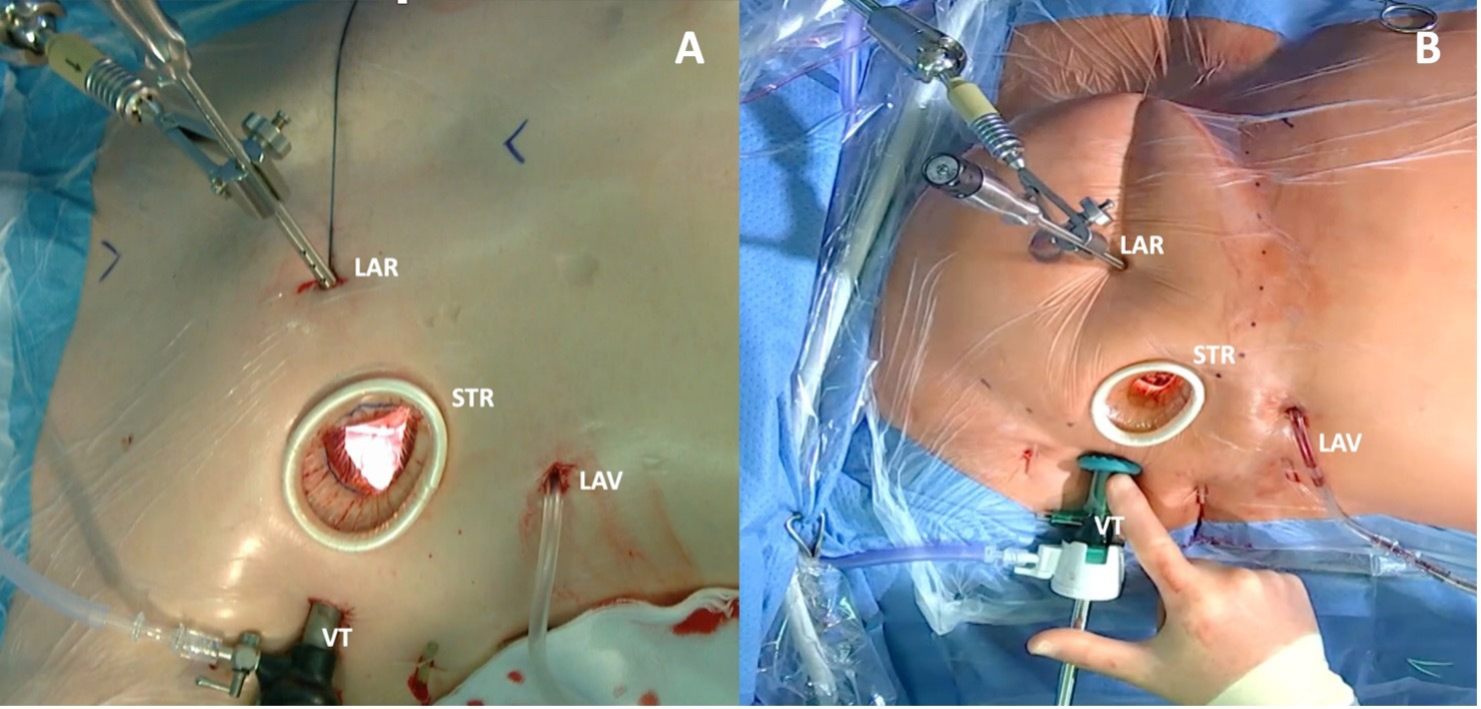
**Minimally invasive thoracic surgery setup**. (A) Periareolar incision: a 3 cm small convex incision that straddles the right areolar border is performed. (B) Right lateral mini-thoracotomy: a small 3–4 cm right lateral incision is performed through the fourth intercostal space. LAR, fixation for the left atrial retractor, placed through a 10 mm port along the fourth intercostal space on the parasternal line; LAV, left atrial vent, placed through the sixth intercostal space; STR, soft tissue retractor; VT, 30° Three-Dimensional (3D) high-definition video thoracoscope, placed into the chest via a 10 mm port through the fourth intercostal space at the right anterior axillary line.

In our center, almost all patients with primary MR are treated with MVr in a minimally invasive setting, with the correction of prolapsing segments and ruptured chordae by the implantation of neochordae using the loop technique and concomitant annuloplasty with a semi-rigid ring to achieve a durable repair [23]. Secondary MR is treated through annuloplasty, preferably with the use of a closed ring to enable a reverse remodeling of the left ventricle [24]. Routinely, a right-sided mini-thoracotomy or a periareolar incision is performed. The MIS setup at our center has been described previously in detail elsewhere [4, 25] and is illustrated in Figs. 1,2.

Prior literature reports excellent outcomes in terms of repair durability, operative complications, long-term mortality, and freedom from reoperation both in the setting of primary and secondary MVD treated through MIS in high-volume centers with specific expertise [4, 26].

Seeburger *et al*. [21] conducted a retrospective study on 1536 consecutive patients who underwent minimally invasive mitral valve surgery (MI-MVS) for MR between 1999 and 2007, whereas 1339 patients underwent MI-MVr. MVr techniques consisted of ring annuloplasty with or without chordae-replacement or Carpentier-type leaflet resection.

The reported 30-day mortality was 2.4% (n = 32). The Kaplan–Meier estimate for survival at 5 years was 82.6% (95% confidence interval (CI): 78.9–85.7%), and the freedom from MV-related reoperation was 96.3% (95% CI: 94.6–97.4%) at 5 years.

Davierwala *et al*. [27] conducted a retrospective study on 3438 patients treated with MI-MVS through a right small thoracotomy approach at the Leipzig Heart Center between 1999 and 2010. Of these, 2829 patients underwent MVr and 609 underwent mitral valve replacement (MVR), resulting in a total repair rate of 81.2%. The reported overall 30-day mortality was 0.8% (n = 23). Overall, the 5- and 10-year mortality rates were 85.7% (± 0.6%) and 71.5% ± 1.2%, respectively. As for the MVr population, survival rates were 87.0% (± 0.7%) and 74.2% (± 1.4%) at 5 and 10 years, respectively. Meanwhile, the freedom from reoperation rates in the same group were 96.6% (± 0.4%) and 92.2% (± 0.9%) at 5 and 10 years, respectively.

McClure *et al*. [28] conducted a retrospective study on a population of 3133 patients who underwent isolated MV surgery from 1996 to 2011. MIS was performed on 1000 patients; of them, 923 were treated through MVr and 77 through MVR. Myxomatous MV disease was the most common affection (86%, n = 860), while intraoperative death was reported as 0.8% (n = 8). Survival rates for the entire population were 93% (± 1%) at 5 years, 86% (± 1%) at 10 years, and 79% (± 3%) at 15 years. The freedom from reoperation in the MVr population was 96% (± 1%) at 5 years, 95% (± 1%) at 10 years, and 90% (± 3%) at 15 years.

Galloway *et al*. [29] conducted a retrospective study on 3057 patients who underwent MVr; of them, 1601 had degenerative MVD and were the object of the study. A total of 1071 patients with degenerative MVD were treated with a MIS approach, and 530 were treated through median sternotomy. An ulterior division into two subgroups was made based on the type of intervention: 712 patients underwent isolated MI-MVr, 223 patients underwent isolated MVr through sternotomy, and 666 patients underwent MVr plus a concomitant cardiac procedure; both MIS and sternotomy were considered in this subgroup. In-hospital mortality was 2.2% in the entire population (36 of 1601), 1.3% for the isolated MI-MVr population (9 of 712), and 1.3% in the group of isolated MVr conducted through median sternotomy (3 of 223). The 8-year freedom from reoperation was 95% (± 1%) in the isolated MI-MVr group and 91% (± 2%) in the isolated MVr sternotomy group (*p* = 0.24). The 8-year freedom from reoperation or severe recurrency of MR was 93% (± 1%) for isolated MI-MVr and 90% (± 2%) for the isolated MVr through sternotomy group (*p* = 0.30). Comparatively, the 8-year freedom from all valve-related complications was 90% (± 2%) in the isolated MI-MVr group and 86% (± 3%) in the isolated MVr through sternotomy group (*p* = 0.14).

Glauber *et al*. [30] conducted a retrospective study of 1604 patients who underwent MI-MVS between 2003 and 2013. Degenerative MVD (70%) was the predominant pathology, followed by functional MVD (12%). MVr was performed in 1137 patients, while MVR was performed in 476 patients. Overall, in-hospital mortality was 1.1% (n = 19), and the repair rate was 95%. Overall survival rates at 1-, 5-, and 10 years were 96.3% (± 0.5%), 88.9% (± 1.1%), and 84.5% (± 1.8%), respectively. Comparatively, the survival rates in the MVr group were 98.5% (± 0.4%) at 1 year, 91.9% (± 1.2%) at 5 years, and 88.0% (± 2.1%) at 10 years. Overall, the freedom from reoperation was 98.6% (± 0.3) at 1 year, 94.7% (± 0.7%) at 5 years, and 91.1% (± 1.7%) at 10 years. Meanwhile, the freedom from reoperation in the MVr group was 98.4% (± 0.4%) at 1 year, 94.8% (± 0.9%) at 5 years, and 93.6% (± 1.1%) at 10 years.

Moscarelli *et al*. [31] conducted a retrospective study on a population of 51 consecutive patients with severe secondary MR, left ventricular ejection fraction <40%, and persistent symptoms despite optimal medical therapy, who underwent minimally invasive MVr with restrictive annuloplasty. The mean duration of follow-up was 33.5 ± 16.8 months, with a 94% survival probability and 97% freedom from at least moderate MR or reintervention identified at the median follow-up.

D’Alfonso *et al*. [32] conducted a retrospective study on 179 patients who underwent MI-MVr between 1999 and 2010. Degenerative MVD was the most represented pathogenesis (95%, n = 170); the remaining nine patients (5.0%) had endocarditis. No in-hospital deaths were reported. The 10-year follow-up reported a survival rate of 98.7% (± 1.2%) and a freedom from reoperation rate of 98.5% (± 1.1%).

A summary of the reported long-term outcomes is presented in Table 1 (Ref. [21, 27, 28, 29, 30, 31, 32]).

**Table 1.** **Summary of reported long-term outcomes of MI-MVr**.

Author, year	Population	Mitral valve disease	Outcome timing	Mortality	Freedom from reoperation
Seeburger *et al*. [21], 2008	1339 patients	92.3% degenerative	5 years	82.6% survival	96.3%
		7.7% functional ischemic			
Davierwala *et al*. [27], 2013	2829 patients	/	5 and 10 years	Survival rates for the repair population:	For the repair population:
				87% (± 0.7%) at 5 years	96.6% (± 0.4%) at 5 years
				74.2% (± 1.4%) at 10 years	92.9% (± 0.9%) at 10 years
McClure *et al*. [28], 2013	Total: 1000 MVr: 923 and MVR: 77	For the total population:	5, 10, and 15 years	Survival rates for the total population:	For the repair population:
		86% myxomatous		93% (± 1%) at 5 years	96% (± 1%) at 5 years
		6% rheumatic		86% (± 1%) at 10 years	95% (± 1%) at 10 years
		4% dilatative cardiomyopathy		79% (± 3%) at 15 years	90% (± 3%) at 15 years
		3% endocarditis			
		0.4% functional ischemic			
Galloway *et al*. [29], 2009	712 patients who underwent isolated MI-MVr	Degenerative MR	8 years	/	95% (± 1%)
					93% (± 1%) freedom from reoperation OR severe recurrent insufficiency
					90% (± 2%) freedom from all valve-related complications
Glauber *et al*. [30], 2015	Total: 1604 MVr: 1137 and MVR: 467	For the total population:	1, 5, and 10 years	Survival rates for the repair population:	For the repair population:
		70% degenerative		98.5% (± 0.4%) at 1 year	98.4% (± 0.4%) at 1 year
		12% functional		91.9% (± 1.2%) at 5 years	94.8% (± 0.9%) at 5 years
		9.4% rheumatic		88.0% (± 2.1%) at 10 years	93.6% (± 1.1%) at 10 years
		5% endocarditis			
		3.2% prosthetic dysfunction			
Moscarelli *et al*. [31], 2021	54 patients	Functional	4 years	90% survival	89%
D’Alfonso *et al*. [32], 2012	179 patients	95% degenerative	10 years	98.7% (± 1.2%)	98.5% (± 1.1%)
		5% endocarditis			

MI-MVr, minimally invasive mitral valve repair; MR, mitral regurgitation; MVR, mitral valve replacement; MVr, mitral valve repair.

## 2. Special Challenges in Minimally Invasive Mitral Valve Surgery

### 2.1 Mitral Annulus Disjunction and Myxomatous Degeneration of Mitral Valve

Mitral annulus disjunction (MAD), first described by Hutchins *et al*. [33], is a separation between the atrial wall–MV junction and the left ventricular attachment. MAD is commonly associated with mitral valve prolapse (MVP) and sudden cardiac death (SCD) [34] and is characterized by the “curling” phenomenon: an unusual systolic motion of the posterior mitral ring on the adjacent myocardium identifiable in echography [35].

MAD and systolic curling account for hypermobility of the MV apparatus and systolic stretch of the myocardium closely linked to the valve, leading to ventricular fibrosis and, consequently, ventricular arrhythmias [35].

Left ventricle fibrosis at the level of papillary muscles and inferobasal wall, myxomatous MV, MAD, and systolic curling define the entity of “arrhythmic MVP” [36].

Arrhythmic MVP is currently an underestimated cause of arrhythmic SCD, mostly in young female adults [36], with an estimated rate of concurrent SCD that ranges in prospective follow-up studies from 0.2%/year to 0.4%/year [37]. The association between MAD and myxomatous disease of the MV, both in the setting of arrhythmic MVP and non-arrhythmic MVP, is a common finding [38].

Hutchins *et al*. [33] hypothesized that MAD could trigger mechanical stress on the leaflets, leading to myxomatous degeneration because of excessive mobility of the MV apparatus. Basso *et al*. [36] confirmed this hypothesis, stating that MAD is the cause of systolic curling motion and, thus, a hypermobility that represents the basis for the paradoxical increase in myxomatous disease of MV leaflets. The markedly myxomatous valve, often referred to as “Barlow’s disease”, is associated with a higher risk of SCD [39]. Multiple authors have studied the pathophysiology of ventricular arrhythmias and SCD in the setting of arrhythmic MVP. Sriram *et al*. [40] suggested that ventricular arrhythmias could be triggered by the concomitant traction on papillary muscles, endocardial friction lesions, coronary microembolism from platelet–fibrin aggregates adjacent to the prolapsing MV leaflet, transient ischemia due to mechanical alterations in coronary blood flow, and increased autonomic tone.

Dejgaard *et al*. [34] hypothesized that MAD represents a clear risk marker of SCD through being a precursor of degenerative MVD and MVP. Therefore, the disjunctive areas along the mitral annulus may, in fact, represent weak spots that are vulnerable to long-standing mechanical stress and development of MVP, which, ultimately, leads to degeneration of the MV apparatus.

**Fig. 3.**
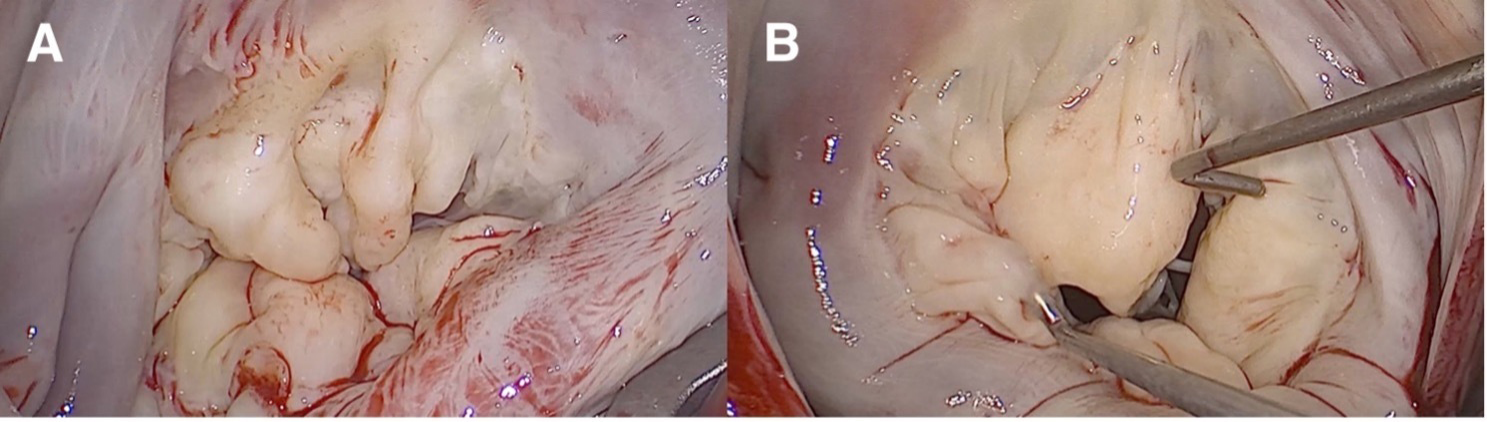
**Vision of a myxomatous Barlow’s mitral valve through a minimally invasive approach**. (A) Appearance of a myxomatous mitral valve. A1, P1, and P2 scallops are prolapsed, and P3 scallop is flailed. There is a cleft between the P1 and P2 scallops as well as between the P2 and P3 scallops with concomitant annulus dilatation. The patient underwent neochordae implantation on P1, P2, and P3, and cleft closure between the P1/P2 scallops and P2/P3 scallops. The implantation of a semi-rigid closed ring supported the repair. (B) Appearance of a myxomatous mitral valve. A2, A3, P2, and P3 scallops are prolapsed, and there is a cleft between the P2 and P3 scallops with concomitant annulus dilatation. The patient was treated with neochordae implantation on A2, A3, P2, and P3, as well as through a cleft closure between the P2 and P3 scallops. The implantation of a semi-rigid closed ring supported the repair.

Basso *et al*. [36] suggest that prolapsing leaflets in MVP lead to myocardial stretch and fibrosis in the inferobasal left ventricular wall and papillary muscles, which act as a trigger for electrical instability. Moreover, MVr represents a successful treatment of ventricular arrhythmias in arrhythmic MVP [41, 42, 43], likely through relieving the mechanical stretch that acts as a trigger on the substrate of myocardial fibrosis. Meanwhile, MVr in the setting of MAD can be challenging, especially in patients with Barlow’s disease, which is associated with less favorable long-term results and higher rates of reintervention when compared to MVr for other MVDs [44]. Indeed, an advanced myxomatous MVD is challenging to face due to the high complexity of the three-dimensional (3D) anatomy of the diseased valve (Fig. 3), which often presents with thick fibrotic and even calcified MV apparatus that may not be amenable to repair [45].

When MAD is present, the number of prolapsing segments can be proportional to the shift of the mitral annulus [44]. Patients with marked leaflet redundancy and excessive posterior leaflet height (>30 mm) [46] have a higher risk of left ventricular outflow tract obstruction due to systolic anterior movement (SAM) of the anterior leaflet [47]. In these high-risk patients, SAM may be prevented by using a concomitant sliding plasty technique and large annuloplasty rings [48, 49].

Minimally invasive access could appear similar to an ulterior challenge in this scenario. However, the minimally invasive approach did not appear to be inferior to MVr conducted through a conventional approach in treating Barlow’s disease and bileaflet prolapse [50], with good early and long-term results indicated when performed in specialized centers [51].

The MIS approach guarantees the complete reproducibility of the optimal standard-of-care results for MVr; however, referral to a center and surgeon with extensive experience in MI-MVr is recommended, given the complex anatomy of diseased MV and the noted steep learning curve associated with MIS [52, 53]. A multitude of repair strategies, firstly proposed via sternotomy, can be successfully employed through the MIS approach. A summary of repair strategies for Barlow’s disease is available in Table 2 (Ref. [44, 47, 49, 51, 54, 55, 56, 57, 58, 59, 60, 61, 62]).

**Table 2.** **Summary of repair strategies for Barlow’s disease**.

Author, year	Population (patients)	Surgical approach	Repair strategy
Eriksson *et al*. [44], 2005	67	MS	Multiple horizontal mattress sutures, P2 resection with chordae reattachment and annuloplasty
Maisano *et al*. [47], 2000	82	MS	Edge-to-edge technique
			with concomitant annuloplasty
Lapenna *et al*. [54], 2005	48	MI	Edge-to-edge technique with concomitant annuloplasty
Adams *et al*. [49], 2006	67	20% (n = 13) MI	Large annuloplasty ring
		80% (n = 54) MS	
Borger *et al*. [51], 2014	145	MI	Loop technique
			with concomitant annuloplasty with a large ring
Quigley [55], 2005	47	MS	Elliptical excision at the base of AML with concomitant annuloplasty
Barlow *et al*. [56], 2003	60	MS	Resection and plication of the prolapsed leaflet
Lawrie *et al*. [57], 2009	61	MS	Multiple stay stitches of 2-0 polypropylene, placed with concomitant annuloplasty
Ben Zekry *et al*. [58], 2015	24	MS	Ring-only repair
Miura *et al*. [59], 2015	12	50% (n = 6) MI	Triangular resection, chordal replacement, and annuloplasty
		50% (n = 6) MS	
Fasol and Mahdjoobian [60], 2002	37	MS	Complete resection of the middle scallop of PML, sliding and folding plasty of the remaining lateral scoops, combined with a triangular resection of AML with concomitant annuloplasty
da Rocha E Silva *et al*. [61], 2015	120	MI	68 patients loop technique
			44 patients edge-to-edge technique
			8 patients, both the loop technique and the edge-to-edge technique
Muneretto *et al*. [62], 2015	50	MI	Resection of PML with sliding at the level of P2 and chordal replacement or isolated artificial neochordae implantation

AML, anterior mitral leaflet; MI, minimally invasive; MS, median sternotomy; PML, posterior mitral leaflet.

The concomitant treatment of the tricuspid valve, a left atrial or biatrial ablation, or additional closure of atrial septal defects does not represent a contraindication for a MIS approach, even when the MV repair is considered complex and time-consuming.

**Fig. 4.**
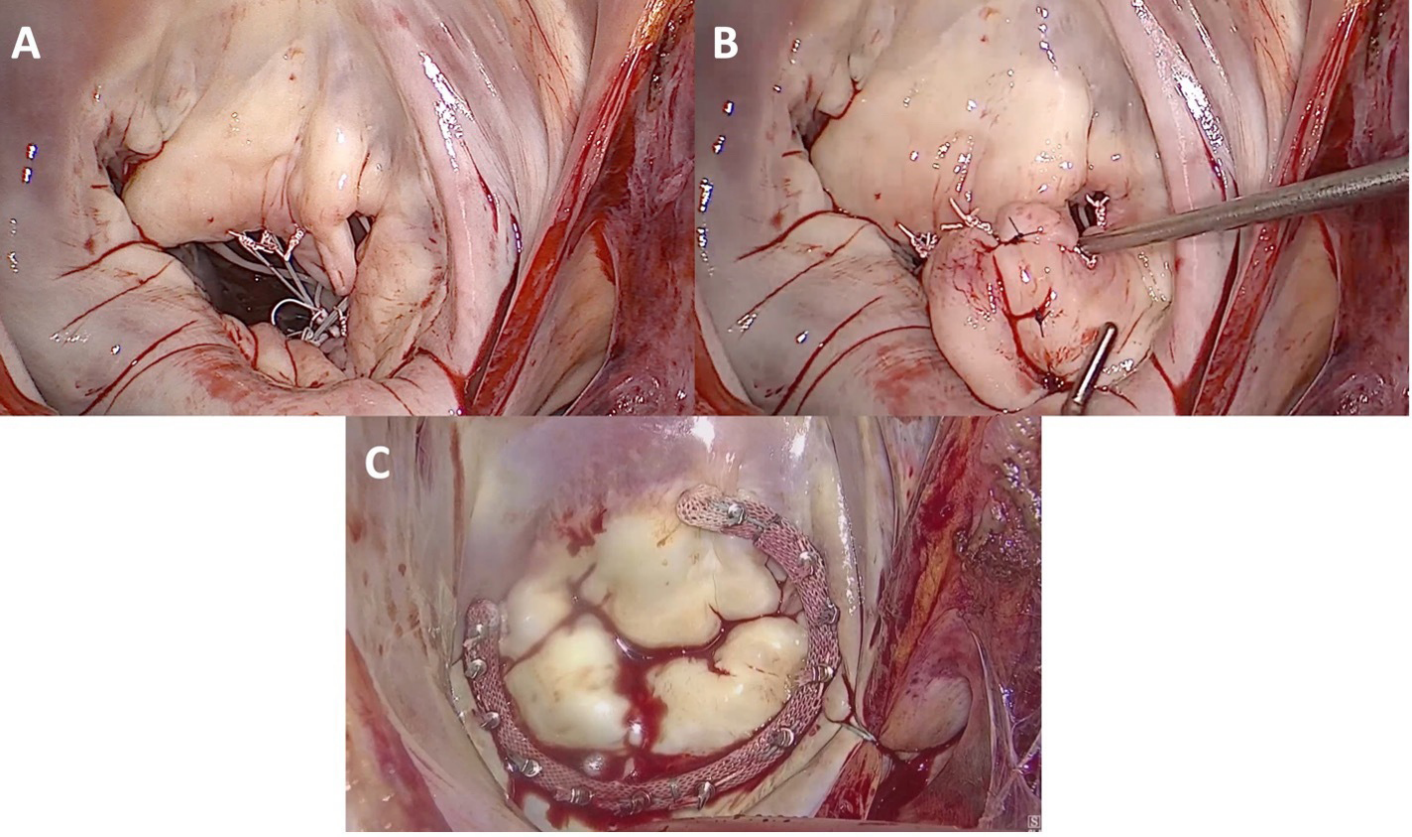
**Minimally invasive mitral valve repair of a mitral valve affected by Barlow’s disease**. (A) Repair of the mitral valve using neochordae and the loop technique on both prolapsing leaflets. The implantation of a semi-rigid closed ring supported the repair. (B) Cleft closure between P1, P2, and P3 scallops in the posterior mitral leaflet. The implantation of a semi-rigid closed ring supported the repair. (C) Annuloplasty with an open, semi-rigid, saddle-shaped ring.

We routinely use the loop technique to correct the prolapsing or flail segment, with attention to patients at high risk of SAM, where the posterior leaflet needs to be corrected aggressively to achieve a posteriorly lying closure line. All patients undergo annuloplasty with a semi-rigid ring (Fig. 4).

### 2.2 Mitral Valve Endocarditis

Infective endocarditis (IE) is estimated to affect 3 to 10 patients per 100,000 per year, with a significant increase in the incidence trend observed over the last thirty years [63]. The current 2023 ESC Guidelines for the management of endocarditis [64] emphasize the importance of early surgical treatment of IE, affirming that a surgical approach may yield a survival advantage of up to 20% within the first year. The main reasons for surgery in the setting of acute IE are represented by heart failure, uncontrolled infection, and prevention of septic embolization [64].

A careful selection of patients with mitral valve infective endocarditis (MVIE) is crucial, as this patient selection enables the performance of MVr whenever feasible, resulting in lower hospital mortality, improved long-term survival, and freedom from disease recurrence compared to MVR with a valvular prosthesis [65, 66].

The approach to minimize surgical trauma through MI-MVr may be particularly useful in surgical treatment for MVIE, as this approach reduces the risk of infections carried by sternotomy and its attendant morbidity, as well as the risk of redo procedures [66, 67]. This approach is also associated with an accelerated recovery in high-risk patients [68], which enhances their comfort, wound healing, and cosmetic results, ultimately leading to a better overall recovery and improved outcomes compared to median sternotomy [69].

**Fig. 5.**
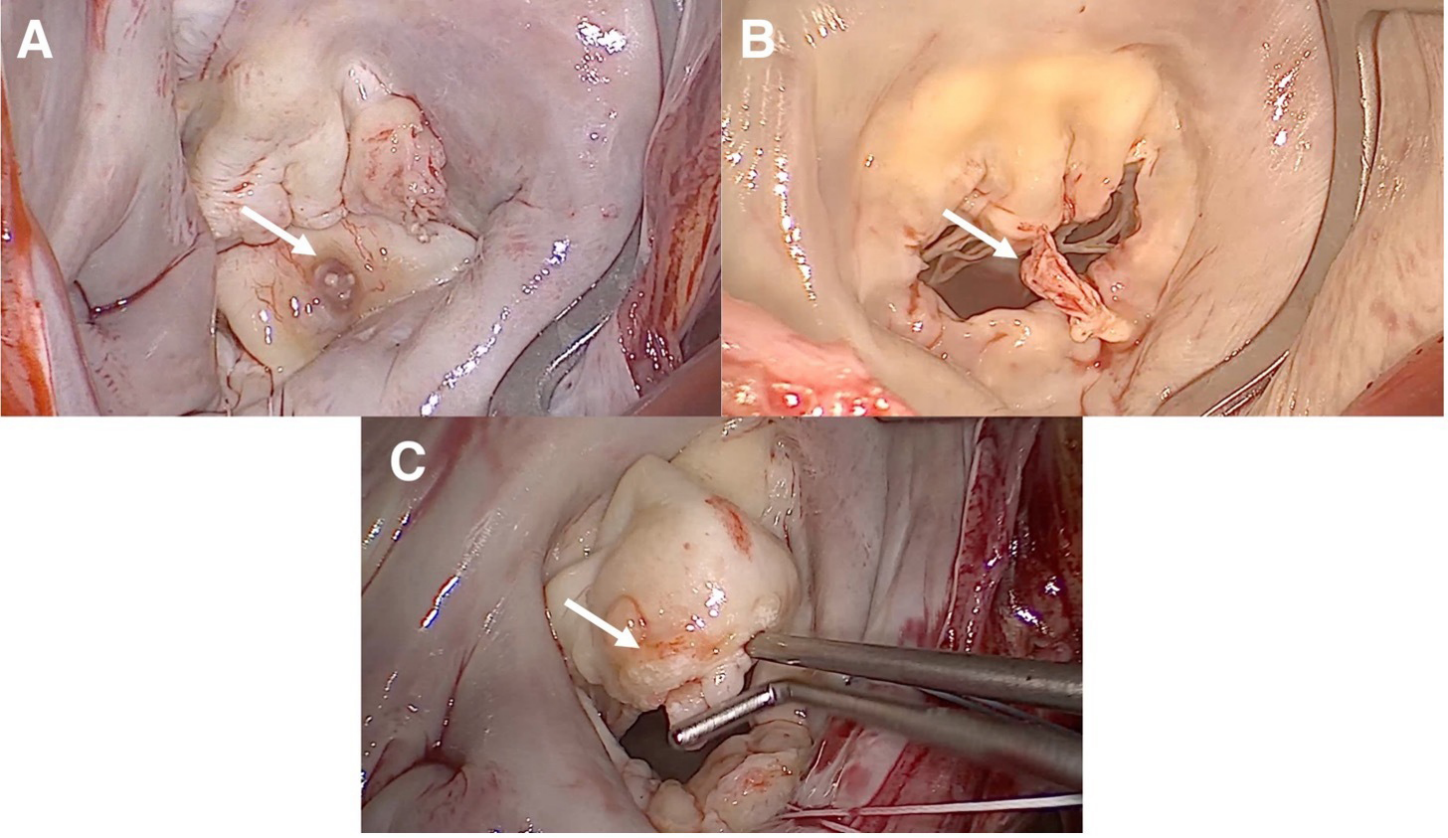
**Images of an endocarditic mitral valve through a minimally invasive approach**. (A) Central vegetation is located on the posterior leaflet. The patient was treated with a quadrangular resection of the P2 scallop, P3 scallop, and A3 scallop, posterior commisuroplasty, neochordae implantation on the P2 scallop, sliding between the P1 and P3 scallops, and implantation of a semi-rigid, closed annuloplasty ring. (B) Large mobile vegetation on the free margin of the posterior leaflet. The patient was treated with resection of the vegetations, cleft closure between the P2 and P3 scallops, neochordae implantation on the A2 scallop, and implantation of a semi-rigid closed annuloplasty ring. (C) Vegetation of the anterior leaflet and chordae tendinae. The patient underwent mitral valve replacement.

Conversely, the limited access and reduced visibility of the MIS setup represent a challenge in the treatment of IE [65], with a steep learning curve, especially in complex cases of valve reconstruction [69]. The appearance of MV endocarditis, as observed through a MIS setup, is illustrated in Fig. 5.

Large vegetations, abscesses, valve destruction, severe involvement of the mitral annulus, participation in the aortomitral continuity, or concomitant mitral annulus calcification (MAC) represent a challenge in annular reconstruction and often require a more extensive surgical intervention, best achieved through median sternotomy [70, 71].

Thus, careful patient selection is fundamental to identifying patients who clearly benefit from a minimally invasive approach [67]. Patients with MVIE that does not extend to the intervalvular fibrous body, aortic valve, or peri-annular tissue are the ideal candidates for an MI-Mvr. Hence, the trend in performing early surgery for native MVIE is another argument in favor of the minimally invasive approach, considering that the disease in this setting is more likely to be confined to the MV [69]. Contrarily, the involvement of the mitral annulus represents a more advanced disease state associated with a worse prognosis and is technically much more demanding to treat, especially in a minimally invasive setup [71]. A redo setting or extensive mitral annular calcification are also anatomical factors that may complicate the complete debridement and excision of all infected tissue, thus representing relative contraindications that should be considered [67].

Recent studies present positive results in patients with MVIE treated using a minimally invasive approach. Indeed, Folkmann *et al*. [67] conducted a retrospective single-center study on 92 patients who were treated using MI-MVr for isolated MVIE. Folkmann *et al*. [67] reported a successful repair rate of 24% (n = 22), a 30-day mortality rate of 9.8% (n = 9), a 1-year survival rate of 77.7% (± 4.4%), and a freedom from reoperation due to endocarditis of 93.3% (± 2.1%). Meanwhile, Barbero *et al*. [68] conducted a retrospective study on 35 patients undergoing MI-MVS for IE; 20% (n = 7) of patients were treated through MVr. Barbero *et al*. [68] reported an overall 30-day mortality of 11.4% (n = 4), with no neurological or vascular complications, and only one reoperation for prosthesis IE relapse occurring 37 days after the initial procedure. The overall survival rate at 1 and 5 years was 83%; the freedom from reoperation and/or recurrence of IE at 1 and 5 years was 97%.

Franz *et al*. [70] conducted a case–control study comparing a group of 75 patients undergoing MIC for MVIE with a group of patients undergoing MIC MV for other reasons (n = 862). Here, Franz *et al*. [70] reported a 30-day mortality rate of 5% (n = 4) in the IE group and of 2% (n = 18) in the non-IE group (*p* = 0.24). Meanwhile, no other significant differences were observed between the two groups in terms of postoperative complications.

Kofler *et al*. [71] compared clinical outcomes between MIS and median sternotomy in patients with native MVIE, conducting a one-to-one nearest neighbor propensity score matching that resulted in a population of 39 matched pairs. Kofler *et al*. [71] reported a shorter overall operative time in the MIS group, as well as an association with fewer transfusions, shorter ventilation times, and a lower rate of reintubation after extubation, resulting in a quicker overall recovery. The 30-day mortality was identical (10.3%, n = 4 in both groups; *p* = 0.375). Kaplan–Meier curves showed a similar survival (*p* = 0.970) during a median follow-up of 3.5 years. Freedom from reoperation was significantly higher in the MIS group (*p* = 0.019), with no patients in the MIS group requiring reoperation, compared to six patients in the median sternotomy group who needed reintervention.

The repair techniques conducted in our center in the setting of MVIE are listed in Table 3 (Ref. [4]). The repair is usually supported by the implantation of a semi-rigid annuloplasty ring [66].

**Table 3.** **Repair techniques in the setting of mitral valve infective endocarditis**.

Lesion	Repair technique
Perforation or leaflet defects	Debridement of vegetations and sequential pericardial patch closure
Annular abscess	Excision of the infected lesions
Atrio-ventricular or atrio-valvular junction disruption	Reconstruction with pericardium or sliding atrium technique
Limited posterior leaflet involvement	Triangular resection with sliding plasty
Anterior leaflet chordae rupture	Secondary chordae transposition or implantation of neochordae
	Leaflet continuity restoration with annular plication or the sliding leaflet technique
Posterior leaflet/commissure chordae rupture	Triangular or quadrangular resection

Adapted from Van Praet *et al*. [4] “Minimally Invasive Surgical Mitral Valve Repair: State of The Art Review.”

### 2.3 Mitral Annulus Calcification

MAC is a chronic degenerative process of the fibrous support structure of the MV, with a reported prevalence ranging from 8% to 15%, which increases to up to 40% in individuals aged 70 years or older [72, 73]. Although MAC was initially considered a passive, degenerative, age-related process, MAC is now recognized as a tightly regulated process that exhibits similarities with medial and atherosclerotic cardiovascular calcification [72]. MAC occurs principally in female and older patients with multiple comorbidities and has a strong association with cardiovascular risk factors [72, 74, 75]. The Framingham Heart Study [76] reported that MAC is an independent predictor of cardiovascular risk, with an increased risk of incident cardiovascular disease, cardiovascular death, and all-cause death. Moreover, the Framingham Heart Study [76] estimated a 10% increased risk for cardiovascular disease for every 1 mm increase in MAC. Carotid atherosclerotic disease, peripheral artery disease, and coronary artery disease are all strongly associated with MAC and the pathogenesis of atherosclerosis [72]. Prevalence, severity, and incidence of MAC are associated with increased MV stress conditions such as hypertension, aortic stenosis, hypertrophic cardiomyopathy, and MVP [72].

MAC involves dystrophic calcium deposition between the ventricular myocardium and leaflet insertion, further calcium aggregates from bars of calcium that may lead to mitral dysfunction [74]. MAC usually affects the posterior annulus with progressive disease calcification that can extend into the rest of the annulus and leaflet tissue [74]. However, leaflet tips are usually not restricted, and commissural fusion is absent [75].

MAC does not necessarily affect the valve functioning, but can be associated with MR or mitral stenosis (MS) through different mechanisms: the rigidity that distorts the valve apparatus and interferes with ring contractility can produce a traction on the chordae and leaflet elevation, leading to MR; the extension of calcium into the valve leaflet, which results in fixation of the leaflet and reduction of the MV orifice area, leads instead to MS.

However, MAC often remains asymptomatic and is, in most cases, an incidental finding; however, if MAC progresses, symptoms of valve dysfunction may occur. Endocarditis, thrombo-embolic events, cardiomyopathy, and congestive heart failure are clinical implications that can develop in concomitance with MAC [72, 74].

Furthermore, up to 20% of patients who undergo MV surgery have some degree of MAC and thus a higher risk of mortality and morbidities compared to patients in whom MAC is absent [75]. MAC influences the outcome of surgery [72]; therefore, surgery is only indicated in the known presence of MAC when there is a relevant degree of valve dysfunction [73, 74, 77].

**Fig. 6.**
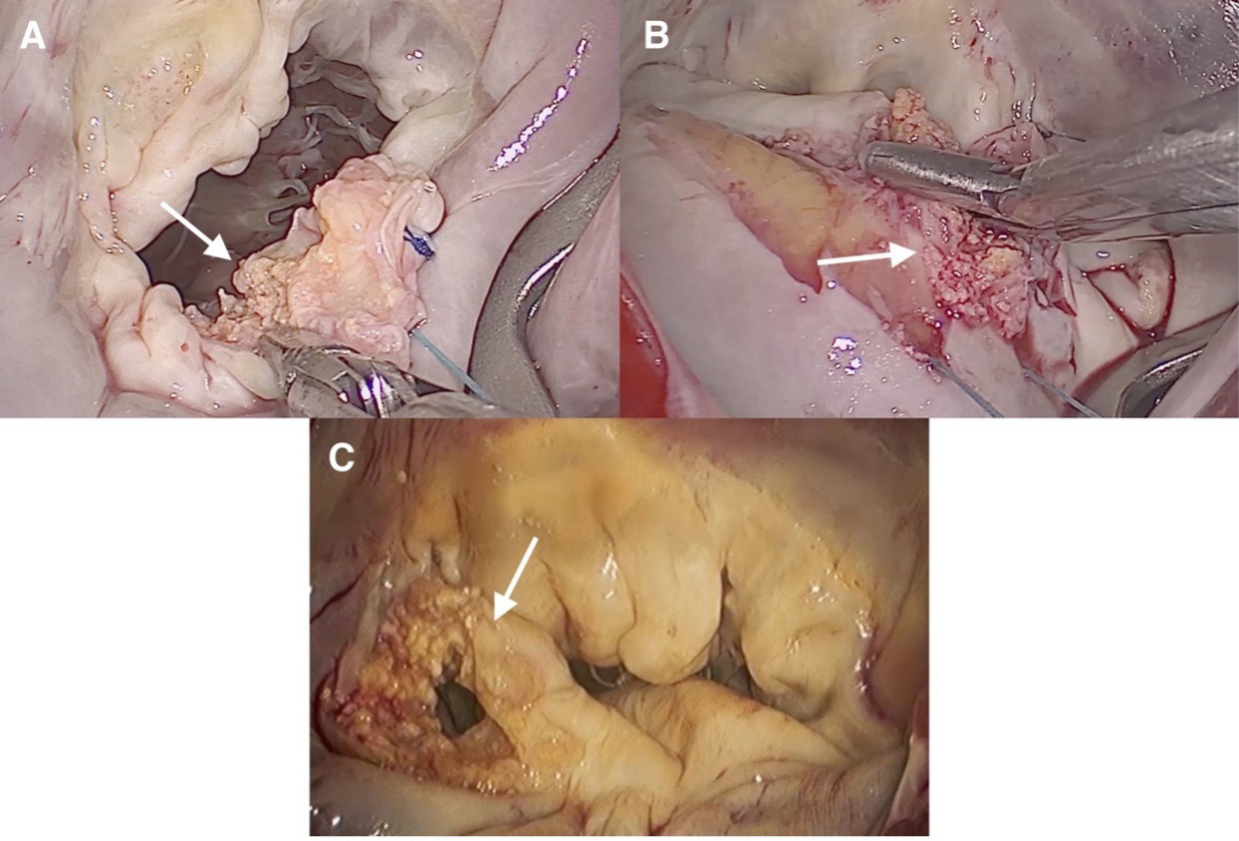
**Images of MAC through a minimally invasive approach**. (A) Calcification of the P2 scallop. The patient was treated with resection of the calcification, closure of the cleft between the P2 and P3 scallops, neochordae implantation on the P2 scallop, and implantation of a semi-rigid open, saddle-shaped annuloplasty ring. (B) Calcification of the posterior annulus. The patient underwent treatment that included resection of the calcifications, neochordae implantation on the P2 scallop, triangular resection of the P3 scallop, annulus reconstruction, and implantation of a semi-rigid, closed annuloplasty ring. (C) Calcification of the P1 scallop. The patient underwent resection of the calcification, neochordae implantation on the P2 scallop, and implantation of a semi-rigid, closed annuloplasty ring. MAC, mitral annulus calcification.

The challenges for the surgeon facing MAC are primarily driven by the characteristics of the patient, which are typically those of old age, accompanied by multiple comorbidities and cardiovascular risk factors [75], and in part by the intrinsic technical difficulty of surgically treating the disease. The main challenges during surgery are related to the difficulty in passing sutures through calcification and, consequently, in accurately placing a prosthetic valve when MVR is performed. The presence of MAC also brings a greater risk of circumflex artery injury or occlusion, atrioventricular dehiscence, and bleeding [74]. However, a minimally invasive approach may be feasible and safe in a select group of patients with limited MAC and should be preferred due to its previously discussed advantages. The MIS visualization of a MAC is shown in Fig. 6.

Strategies to address MV surgery in patients with MAC are categorized into two different approaches: the “resect approach” and the “respect approach” [73, 78], which are undertaken both in the setting of MIS and median sternotomy.

The “resect approach” involves the decalcification of the annulus and its subsequent reconstruction. This approach is mainly used in the context of MVr [73], but can also be conducted as a first step before MVR. Debridement of calcium before MVR may allow an easier placement of sutures, a more “natural” appearance of the prosthetic valve for size and position, and a lower risk of paravalvular leaks. However, the “resect approach” is technically challenging and carries the risk of atrioventricular disruption [73] when an excessive debridement of the calcified annulus is performed. Atrioventricular disruption is a catastrophic complication that represents the “worst-case-scenario” during MI-MVr and eventually requires conversion to sternotomy to be successfully treated.

The “respect approach” avoids annular decalcification and allows for simpler and shorter surgery with a lower risk of atrioventricular groove disruption. Nonetheless, the “respect approach” is more likely to lead to paravalvular leaks and injuries to the circumflex artery, conduction system, and coronary sinus [73, 74, 75, 79].

Most patients with non-severe MAC benefit from calcium debridement, annular reconstruction, and MVr. However, in the presence of a severe MAC, with the involvement of more than one-third of the annular circumference [80], MVR with a “respect approach” is usually preferred. A “resect approach” followed by MVR in the context of severe MAC has, however, been proposed by multiple authors, utilizing ultrasonic debridement of calcifications before MVR [81, 82, 83, 84].

The “resect approach” in the context of MVr has been proposed by Carpentier *et al*. [80], who performed an annulus decalcification followed by reconstruction in a population of 68 patients, with a successful repair in 67 patients.

Feindel *et al*. [85] proposed a debridement of annular calcification followed by the creation of a new annulus through a pericardial patch in 54 patients, with a 5-year survival rate of 73% (± 7%) and a 5-year freedom from reoperation rate of 89% (± 6%). Survival rate at 7 years was 93.1% (± 7.5%) and freedom from reoperation at 9 years of 87.1% (± 16.7%).

Multiple authors have performed the “respect approach” in the setting of MVR. Akansel *et al*. [78] conducted MI-MVr in a patient with noncircumferential moderate MAC performing MVr with a “respect approach”. Akansel *et al*. [78] avoided manipulation of the MAC and conducted a repair with neochordae loops implantation and positioning of a posterior band cut in its midpoint to prevent the MAC zone of the annulus. MI-MVr with partial annuloplasty can be safely performed in patients with noncircumferential MAC, avoiding calcium debridement [78].

## 3. Conclusions

MI-MVr represents an excellent surgical option in complex MVD, such as endocarditis, MAC, and MAD with myxomatous degeneration of the MV. When performed in high-volume centers by experienced surgeons, this approach offers a fast recovery and higher patient comfort without compromising surgical efficacy or patient safety. Therefore, careful patient selection, proper surgeon training, and high-volume centers with specific expertise in minimally invasive mitral surgery are key elements in achieving optimal outcomes in these challenging cases.

## 4. Limitations

This narrative review is based on the currently published literature, which primarily consists of observational and comparative studies. Additionally, as a narrative review, this paper does not include a quantitative synthesis or meta-analysis. These factors represent inherent limitations that may affect the strength and generalizability of the conclusions.

## Abbreviations

IE, Infective Endocarditis; MAC, Mitral Annulus Calcification; MAD, Mitral Annulus Disjunction; MI-MVr, Minimally Invasive Mitral Valve Repair; MI-MVS, Minimally Invasive Mitral Valve Surgery; MIS, Minimally Invasive Surgery; MR, Mitral Regurgitation; MV, Mitral Valve; MVD, Mitral Valve Disease; MVIE, Mitral Valve Infective Endocarditis; MVP, Mitral Valve Prolapse; MVr, Mitral Valve Repair; MVR, Mitral Valve Replacement; SAM, Systolic Anterior Movement; SCD, Sudden Cardiac Death.
